# Experimental Selection for *Drosophila* Survival in Extremely Low O_2_ Environment

**DOI:** 10.1371/journal.pone.0000490

**Published:** 2007-05-30

**Authors:** Dan Zhou, Jin Xue, Jianming Chen, Patrick Morcillo, J. David Lambert, Kevin P. White, Gabriel G. Haddad

**Affiliations:** 1 Departments of Pediatrics, Section of Respiratory Medicine, and Neuroscience, University of California San Diego, La Jolla, California, United States of America; 2 Department of Immunology, The Scripps Research Institute, La Jolla, California, United States of America; 3 Department of Cell Biology, Albert Einstein College of Medicine of Yeshiva University, Bronx, New York, United States of America; 4 Department of Biology, University of Rochester, Rochester, New York, United States of America; 5 Institute for Genomics & Systems Biology and Departments of Human Genetics and Ecology and Evolution, The University of Chicago, Chicago, Illinois, United States of America; 6 The Rady Children's Hospital - San Diego, San Diego, California, United States of America; Massachusetts General Hospital, United States of America

## Abstract

**Background:**

Cellular hypoxia, if severe enough, results usually in injury or cell death. Our research in this area has focused on the molecular mechanisms underlying hypoxic tissue injury to explore strategies to prevent injury or enhance tolerance. The current experiments were designed to determine the genetic basis for adaptation to long term low O_2_ environments.

**Methodology/Principal Findings:**

With long term experimental selection over many generations, we obtained a *Drosophila melanogaster* strain that can live perpetually in extremely low, normally lethal, O_2_ condition (as low as 4% O_2_). This strain shows a dramatic phenotypic divergence from controls, including a decreased recovery time from anoxic stupor, a higher rate of O_2 _consumption in hypoxic conditions, and a decreased body size and mass due to decreased cell number and size. Expression arrays showed that about 4% of the *Drosophila* genome altered in expression and about half of the alteration was down-regulation. The contribution of some altered transcripts to hypoxia tolerance was examined by testing the survival of available corresponding *P*-element insertions (and their excisions) under extremely low O_2_ conditions. We found that down-regulation of several candidate genes including *Best1*, *broad*, *CG7102*, *dunce*, *lin19-like* and *sec6* conferred severe hypoxia tolerance in *Drosophila*.

**Conclusions/Significance:**

We have identified a number of genes that play an important role in the survival of a selected *Drosophila* strain in extremely low O_2_ conditions, selected by decreasing O_2_ availability over many generations. Because of conservation of pathways, we believe that such genes are critical in hypoxia adaptation in physiological or pathological conditions not only in *Drosophila* but also in mammals.

## Introduction

Hypoxia, as a result of disease or high altitude, can have devastating effects. Over the past decade, research in this area has begun to focus on the molecular and genetic mechanisms underlying hypoxic tissue injury or tolerance using different animal models. Some of these studies have also explored strategies by which injury could be either prevented or alleviated and tolerance enhanced [1]. The discovery of the hypoxia-inducible-factor (HIF) and its regulation under low O_2_ conditions in vertebrates and invertebrates has opened major avenues for investigating mechanisms of adaptation and potential strategies for therapy [2]. However, there are mechanisms of hypoxia resistance or adaptation that may not be related to HIF function [3], [4], and the underlying genes are, by and large, unknown.

Our previous studies have demonstrated that *Drosophila melanogaster* is very resistant to *acute* anoxia (no O_2_ in environment for a few hours) and does not suffer tissue injury under such severe conditions [5], [6]. By using different molecular and genetic approaches, including a mutagenesis screen, several genes have been identified that regulate the response of this organism to acute anoxia (i.e., *tps1* [7], *ADAR* [6], [8], and *fau* [9]). However, these previous approaches and experiments were not designed to determine the genetic basis for adaptation to long term (i.e., over many generations) low O_2_ environment, such as occurs when organisms live at high altitude. In the current study, we selected for flies (AF) that can complete all life cycle stages and live perpetually in extremely low O_2_ conditions, levels which are prohibitive and lethal for naïve flies (NF). We then used gene expression profiling to identify the genes whose expression levels were significantly different in the NF versus AF. Previous studies have shown that genes underlying experimentally selected multigenic traits (e.g., geotaxis) can be identified by using expression profiling approaches [10]. Here, we employed this approach to study hypoxia tolerance in *Drosophila* and identified genes that play an important role in this selected phenotype.

## Methods

### Parental *Drosophila melanogaster* isogenic lines

In order to provide allelic variation for laboratory selection, we crossed 27 isogenic *Drosophila melanogaster* lines (kindly provided by Dr. Andrew Davis) which have different recovery times in acute anoxia test [8] as well as different elcosion rates when cultured under hypoxic conditions (Figure S1 and S2). Male and virgin female flies (n = 20) from each of the 27 lines were collected and pooled in a chamber and maintained at room temperature with standard food medium. Embryos from this pooled fly population were collected as F1 and subjected to either long term (over many generations) hypoxia (selection experiment) or normoxia (control experiment). In order to determine the starting O_2_ concentration for hypoxia selection, we tested first the feasibility and tolerance of the F1 progenies of the parental cross to different O_2_ concentrations (i.e., 8, 6, or 4% O_2_). In addition, the tolerance of each parental line to hypoxia was measured by testing survival for each individual line in the hypoxic environments. Percentage eclosion was determined by calculating the ratio of the number of empty pupae to the number of total pupae in each culture vial. The selection was started therefore at 8% O_2_ and this concentration was gradually decreased by 1% each 3 to 5 generations to keep the selection pressure. Six separate populations of F1 were cultured in 6 individual chambers, 3 of them were used for hypoxia selection and 3 others for the control experiments. Embryos, 3^rd^ instar larvae and adult flies were collected from each generation and stored at −80°C for subsequent DNA- or RNA-based analyses. The results presented in the current study were derived from RNA-based expression arrays of the 18^th^ generation in adult fly samples.

### Culture chambers

Population chambers (26 cm×16 cm×16 cm) were specially designed for the hypoxia selection experiments. These chambers were connected to either O_2_ balanced with N_2_ at certain O_2_ concentration (for the hypoxia selection experiments) or to room air (21% O_2_, for the control experiments). The humidity in the chambers was maintained by passing the gas through water prior to going into the chambers. The flow speed was monitored by 565 Glass Tube Flowmeter (Concoa, Virginia Beach, VA), and the O_2_ level within the chamber was monitored with Diamond General 733 Clark Style Electrode (Diamond General Development Corp., Ann Arbor, MI).

### Phenotypic assays

Several phenotypic assays were used to determine the phenotypic differences between AF and NF flies. **1) Response to acute anoxia.** Anoxia testing was performed as described previously [8]. Briefly, groups of 10–15 adult flies (3–6 days old) were used in each test. The time taken by each fly to recover after reoxygenation from 5 minutes anoxic stupor was referred to as recovery time. **2) O_2_ consumption.** Oxygen consumption rates were measured as previously described [5] in both normoxic and hypoxic conditions with minor modifications. Briefly, flies were acclimated for about 30 min before any baseline measurement was taken in normoxia. Baseline O_2_ consumption rate was measured over 30 to 60 min at 21% O_2_, and this was followed by a hypoxic period of 30–60 min (3% O_2_). Six to eight hundred flies were used in each O_2_ consumption measurement. **3) Body weight, wing size and cell number measurements.** Male flies (n = 100) from each generation were collected and weighed. For wing size and cell number measurements, the wings from male flies of each group were collected, flattened on glass slides in a drop of xylene and mounted with Permount. The images of the wings were digitized using an Axiovert 200 M microscope (Carl Zeiss MicroImaging, Inc., Thornwood, NY, USA) with an image capturing Axiovision software program at a magnification of 10× (whole wing, 964 pixels/mm) and 63× (microchaetes, 6000 pixels/mm). The microchaetes from the whole field were overlaid by dots in a new layer using Adobe Photoshop 7.0.1 and counted using the Scion Image Beta 4.0.2 program. The surface area and the total number of pixels in the defined area were calculated and converted into area units (mm^2^). The wing length, width and the pixel density per mm^2^ under each magnification were measured using a Bright Line Hemacytometer Improved Neubauer 1/400 Square mm grid (Reichert, Buffalo, NY).

### cDNA microarray analysis

cDNA microarrays containing 13,061 known or predicted genes of the *D. melanogaster* genome were processed as described previously [10], [11], [12]. These arrays were used to identify genes that were differentially expressed in AF as compared to NF adult flies. Twelve samples from AF flies and 12 samples from NF were included in this analysis. Each sample contained a pool of 20 male and 20 female flies from each culture chamber. Four samples from each of these chambers were collected and analyzed. Total RNA was extracted using TRIzol (Invitrogen, Carlsbad, CA) followed by a clean-up with RNeasy kit (Qiagen, Valencia, CA). Three µg of total RNA from each sample was amplified with an *in vitro* transcription-based strategy using a one-round linear amplification protocol [12], [13]. A common reference design was applied for the hybridizations, and the reference RNA sample was created by total RNA extracted from a balanced pool of 20 male and 20 female flies from each parental line. Microarray images were acquired by GenePix 4000 microarray scanner using GenePix Pro 3 microarray analysis software (Axon Instruments, Sunnyvale, CA, USA). The differences in gene expression were calculated using the ratio of intensity between hypoxia-selected flies and controls. The statistical significance (false discover rate (FDR), *q*-value) and the ratio of the changes in expression was calculated using Significance Analysis of Microarray (SAM) software [14] following LOWESS normalization. The expression fold changes were presented as ratios, if up-regulated, or −1/ratio, if down-regulated. The microarray analysis data can be retrieved using access number GSE4972 in the Gene Expression Ominibus database at http://www.ncbi.nlm.nih.gov/geo.

### *P*-element mutant lines

*P*-element insertion stocks were obtained from *Drosophila* Stock Centers (Bloomington, IN., USA, and Szeged, Hungary) [15]. The presence and the position of *P*-elements was verified by inverse PCR using primers complementary to *P*-element sequences and the effect of the *P*-element insertion on specific gene transcript was verified by using semi-quantitative RT-PCR with specific primers of the target gene (Table S3). The relative expression level of target genes was calculated by normalizing the level of a target amplicon to the level of β-actin in the *P*-element line. The *yw* stock was used as control. These *P*-element insertion lines were cultured at 5% O_2_ for about 21 days to determine whether specific genes, obtained from the microarray analysis, were involved in the tolerance to the severely low O_2_ level (5%), a level that is lethal to NF. The 27 parental lines and the *yw* stock used to generate *P*-element insertion lines had an average eclosion rate of less than 10% (Figure S1). A Chi-squared test was used to determine the statistical significance of the percent survival between the *P*-element lines and controls.

### Excision of the *P*-elements KG08199 and EY10058

To excise the *P*-elements, females of y[1]; P{y[+] w[+]}KG08199/CyO; ry[506] or y[1] w[67c23]; P{w[+mC] y[+mDint2] = EPgy2}Atg7[EY10058] were crossed to males that express Δ2–3 transposase. Male or female progenies possessing both the *P*-element and the transposase were then individually crossed to females or males with the 2^nd^ chromosome balancer, CyO. Several independent excision lines for either *P*-element were established. Precise excision was confirmed by sequencing the PCR product of the genomic region around the *P*-element insertion site. Primers used for PCR were: forward primer: 5′-CTGAATTTTGGAGGCTTTGC-3′; reverse primer: 5′-CTCGAAGTGACGCTTTAGGG-3′, and the primer used for sequencing was 5′-CTCGATGGCGATAGACCAAT-3′.

## Results and Discussion

To initiate the process of long term experimental selection, we first generated a heterogeneous parental population of *Drosophila melanogaster* by pooling 27 wild-type isogenic lines. The inter-parental genetic variability of hypoxia tolerance was determined by measuring 1) the eclosion rate in 5% O_2_, and 2) recovery time from anoxic stupor of individual parental lines. We found significant variation in their eclosion rates that ranged from 0 to 28 percent (4.7% in average) in 5% O_2_ (ANOVA, *p*<0.05, Figure S1) and their recovery time from anoxic stupor ranged from 319 to 515 seconds (ANOVA, *p*<0.0001, Figure S2) which demonstrated significant genetic diversity within the parental lines. In order to determine the level of O_2_ at which we needed to start the selection experiment, F1 embryos of this pooled population were collected and cultured in 1 of 3 separate chambers under different levels of hypoxia (8%, 6% or 4% O_2_). There was a dramatic decrease in the percentage of embryos reaching adult flies in 6% O_2_ (<10%), and no adult flies were actually obtained in 4% O_2_. Under 8% O_2_, the majority of the embryos (>80%) completed their development and reached the adult stage. Therefore, hypoxia selection was initiated at 8% O_2_, and the O_2_ concentration was gradually decreased by ∼1% every 3 to 5 generations to maintain selection pressure. The hypoxia level was first reduced to 7% and then lowered further. By the 13^th^ generation, we obtained flies that were able to complete their development and perpetually live at 5% O_2_. The AF flies showed >50% survival rate during the first generation at 5% O_2_ (the 13^th^ generation), and this survival rate increased to more than 80% in following generations in 5% O_2_. More recently, we have obtained AF flies that can even tolerate 4% of O_2_ perpetually after 32 generations of selection. We hypothesized that this is, at least partially, due to newly occurring mutations or recombination of favorable alleles in the selected population. Our selection paradigm allowed us, therefore, to obtain flies that can complete their development and tolerate perpetually 5% and even 4% O_2_, conditions that are lethal to NF.

To test the hypothesis that AF flies are a result of selection of favorable genetic allelic variants, a subset of embryos obtained from the AF flies at the 18^th^ generation were collected and cultured under *normoxic* condition for several consecutive generations. After 8 generations cultured in normoxia, they were re-introduced to a 5% or 4% O_2_ environment, and again, the majority (>80%) of the flies completed their development and could be maintained in this extreme condition perpetually. This result demonstrated that the selection, indeed, resulted in a heritable trait.

In our experiments, we have pooled equal numbers of parental males and virgin females to provide equal representative progenies from the isogenic lines. It is interesting to note that there were natural differences in the survival of the parental lines to low O_2_ (5%). Whether the inherited tolerance that we obtained in the selected flies derived mostly from the genome of some parental lines, such as DMN6, DMN12 or DMN20, which showed greater hypoxia tolerance, is not known at the current stage.

**Figure 1:**
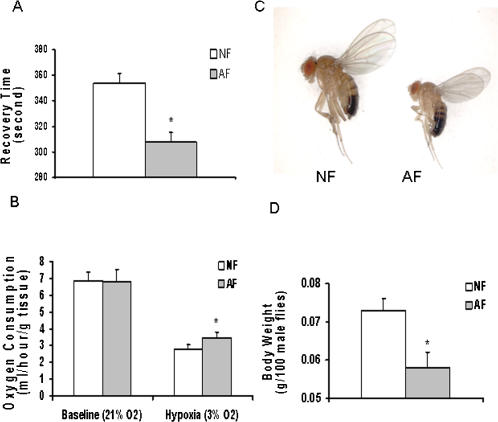
Phenotypic changes following long term hypoxia selection. (A): Shortened recovery time of AF flies from anoxic stupor. Groups of 10 to 15 NF or AF flies were subjected to pure N_2_ for 5 min (anoxia) at room temperature. Recovery time of each fly from the time of anoxic stupor to that of arousal following reintroduction of room air was recorded. A significantly shortened recovery time was found in AF flies (*p*<0.01; NF: n = 166, AF: n = 114). (B): Increased O_2_ consumption rate of AF flies in hypoxic condition. O_2_ consumption rate was measured in a sealed testing jar at room temperature under normoxia (21% O_2_, Baseline) and hypoxia (3% O_2_, Hypoxia), respectively. Groups of 600 to 800 NF or AF flies were used in each test. Although both AF and NF decreased their O_2_ consumption when switched to hypoxic condition, AF flies reduced less than NF (*p*<0.01, n = 5). (C and D): Decreased body size and weight in AF flies. AF flies had decreased body weight and size. Body weight of a group of 100 NF or AF male flies were measured at 16^th^ and 17^th^ generation (n = 6) following hypoxia selection. A significant decrease at body weight was found in AF flies (lower panel, *p*<0.01). Data were presented as mean ± SEM, and the statistical significance was analyzed by student's *t*-test.

**Figure 2:**
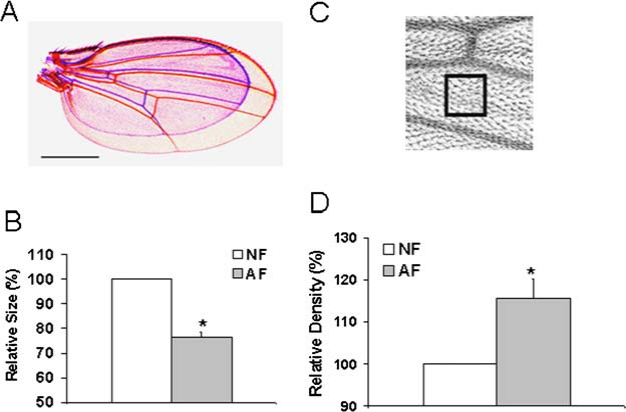
Decreased cell number and size in AF flies. The wing was used as a model organ to determine whether the weight and size reduction in AF flies could be due to either a reduction in cell number, a reduction in cell size (i.e. increase in cell density) or both. Panel A is representative pictures of wings from an AF (blue) and a NF (red) fly (bar = 250 µm). There was more than 20% reduction in wing area in AF (B; n = 16, *p*<0.01), that is not able to be fully compensated by a corresponding increase in cell density (C and D; n = 16, *p*<0.01). Further estimation of the total number of cells in the wing by multiplying the area by the cell density resulted in a total 10.2% cell loss in AF flies. Therefore, the reduction of the fly size is due to decrease of both cell size and cell number. Data were presented as mean ± SEM. The statistical significance was calculated by student's *t*-test.

Several significant phenotypic changes were observed in the AF flies. First, AF flies have shortened recovery time from anoxia-induced stupor. In this test, both AF and NF flies lost coordination and fell to the bottom of the testing jar rapidly after inducing anoxia (0% O_2_) [8]. After a period of 5 minutes of constant anoxia during which flies were motionless, room air (21% O_2_) was bled in the testing jar and both AF and NF recovered. However, the AF flies aroused from anoxic stupor in a much shorter period of time than the NF flies (Figure 1A, p<0.01). Second, both AF and NF flies dramatically decreased their O_2_ consumption (∼1/2 to 1/3) in a low O_2_ environment as compared to room air. This is a well known mechanism used by anoxia-tolerant animals to minimize the mismatch between O_2_ supply (limited O_2_ in the environment) and demands [16]. Interestingly, however, AF flies showed a significantly (p<0.01) higher O_2_ consumption rate in hypoxia, when compared to their NF counterparts (Figure 1B). These results indicated that AF flies have become more resistant to anoxia, and that this may be partly due to an increase in the efficiency of O_2_ utilization. Interestingly, we have observed that these AF flies were more active behaviorally during O_2_ consumption measurements in hypoxia (3% O_2_). Third, there was a significant reduction in body weight and size in the AF flies. In the hypoxia chambers, at 6% and certainly at 5% O_2_ levels, adult flies had significantly decreased body weight and size: the decrease in male body weight was about 25% (Figure 1C and 1D). The size, cell number and density were measured in AF and NF flies to determine if the decrease in body size and weight were due to reduced cell number or/and cell size. As shown in Figure 2A and 2B, the decrease in wing area (as an index of body size) was about 20% in the AF as compared to the NF flies. Further analysis revealed that both cell size and estimated cell number had been significantly reduced by about 15% and 10% respectively (p<0.01, Figure 2C and 2D), demonstrating that hypoxia affected both cell proliferation and growth.

The decrease in body and wing size is interesting from several points of view. First, our previous studies have shown that low O_2_ leads to an arrest in cell cycle activity in *Drosophila* embryos and can have an effect on cell proliferation and embryonic growth [17]. Hence, low O_2_ has a potent impact on a fundamental process that can lead to an important phenotype such as the size of the adult fly. One question that can be raised is whether this decreased size in the AF flies is related to genetic mechanisms that underlie survival at 5% or 4% O_2_. We do not believe that this is the case because the decreased body size immediately reverted to normal size within one generation when AF flies were cultured in normoxia (data not shown), which is very different from the trait of hypoxia tolerance that was shown to be heritable in the AF flies. Regardless of the mechanism, we believe that the AF flies minimize the overall energy demand by reducing their total body mass. In addition, since previous studies have demonstrated that the tracheae increase in diameter after hypoxic exposure over several generations [18], we speculate that the decrease in body size and increase in tracheal diameter would enhance O_2_ delivery. Clearly, this decrease in body size could also be the effect of lower metabolism in low O_2_ conditions. Second, hypoxia not only alters size in invertebrates but also stunts growth in mammals. This decrease in size in mammals has been observed in rodents [19], in humans at high altitude [20], [21], in patients with congenital heart disease with right to left cardiac shunt [22], and in infants with chronic lung disease with insufficient inspired O_2_ concentration [23].

**Figure 3:**
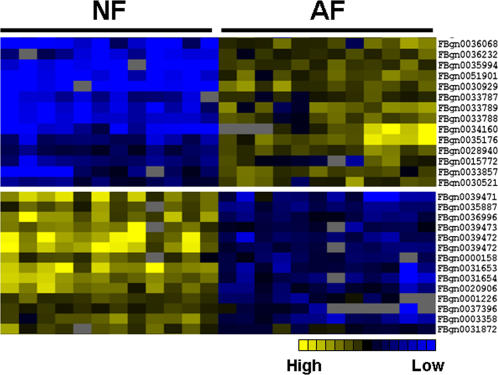
Distinct expression clusters between AF and NF samples revealed by microarrays. cDNA Microarray results were clustered according to the levels of expression of the hypoxia selected and the control cross adult fly samples using GeneCluster [42]. Differentially expressed genes were sharply distinguished between NF and AF flies. Upper panel: a representative subgroup of up-regulated genes. Lower panel: a representative subgroup of down-regulated genes. Yellow color represents relative high levels of expression while blue represents low levels of expression. The brightest color is 1.5-fold or greater differential from the reference black.

The differentially expressed genes in AF samples were identified by comparison of 24 arrays containing 12 replicates of the AF or the NF chambers respectively at the 18^th^ generation (Figure 3). The rationale for using F18 for the microarray analysis is that this generation displayed a phenotypic breakthrough whereby flies had been surviving an O_2_ level for more than 4 generations that was lethal to naïve flies. Direct comparison of the hybridizations between AF and NF samples revealed that 498 genes (∼4.0% of the tested genes) had significantly altered their levels of expression (*q*<0.05, Table S1), with 279 genes being up-regulated and 219 genes down-regulated. Besides, several gene families were found to be significantly altered in the AF flies (*p*<0.05) [24]. For example, antibacterial peptides (12 genes), cytochrome P450 (e.g., cytochrome P450-6 sub-family, 8 genes), and protein kinase C (2 genes) were up-regulated, and proteases (26 genes), phosphatases (5 genes), and triacylglycerol lipases (6 genes) were down-regulated families.

**Figure 4:**
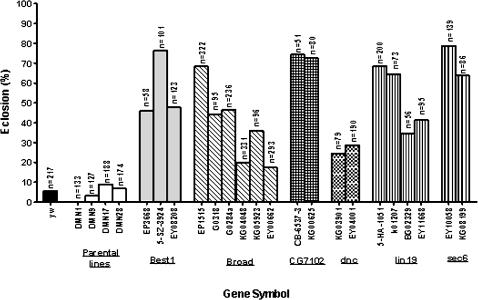
Survival of *P*-element insertion alleles of candidate genes in severe O_2_ environment. Survival of single *P*-element insertion lines for specific candidate genes. Embryos from each *P*-element insertion line were collected and cultured at 5% O_2_ condition. Total and eclosed pupae were counted and the ratio of eclosion for each allele was compared to that of *yw* and NF controls. Each bar represents the average of at least three tests of individual *P*-element insertion lines. The total number of scored pupae was indicated over each bar. The statistical significance was obtained when *p* values were <0.001 (Chi-squared test).

**Figure 5:**
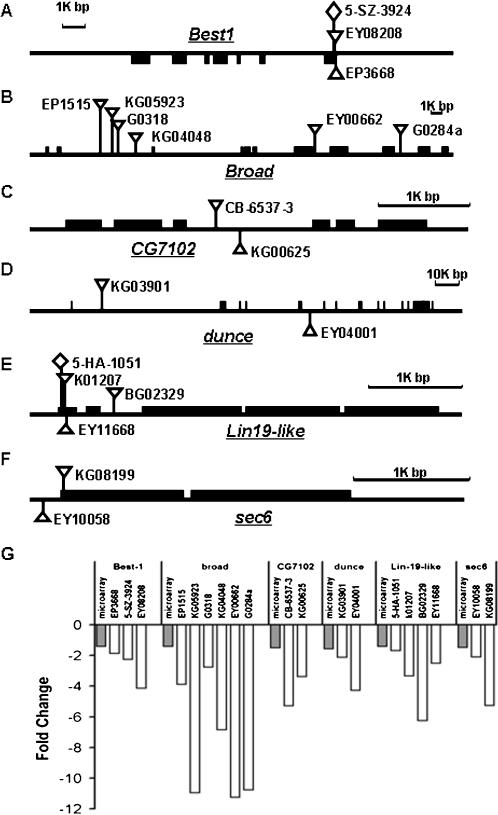
Alteration of target gene expression by *P*-element insertion. (A–F): Genomic localization of *P*-element insertions within or around gene *Best1*, *broad*, *CG7102*, *dunce*, *lin19* and *sec6*. (G): Effect of *P*-element insertion on target gene expression was determined by sq-RT-PCR. Each open-bar represents the mean value of sqRT-PCR of a *P*-element allele for the target gene.

**Figure 6:**
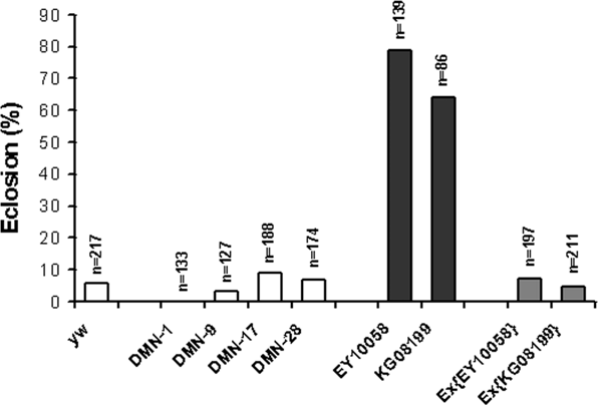
Precise-excision of *P*-elements in genomic region of gene *sec6* reverses hypoxia tolerance. Survival of precise excision lines for *P*-element insertion targeting gene *sec6* was determined in hypoxic condition. Embryos from each precise excision line were collected and cultured in 5% O_2_. Total and eclosed pupae were counted and the ratio of eclosion for each allele was compared to that of *yw*, NF controls and original *P*-element alleles. Each bar represents the average of at least three tests of individual excision line. The total number of scored pupae was indicated over each bar. The statistical significance was obtained when *p* values were <0.001 (Chi-squared test).

Although hypoxia tolerance is likely to be a complex trait which involves coordinated action of many genes, individual gene expression profiling can provide clues about genes that control multigenic traits [10], [25]. However, it is likely that not all of these differentially expressed individual genes are directly responsible for hypoxia tolerance in the selected AF flies. For example, some of these genes might have changed their mRNA levels as a result of ‘hitchhiking’ through genetic linkage, through inadvertent selection for traits other than hypoxia tolerance, or through drift. Thus, only some of these differentially expressed genes are likely to play a role in hypoxia tolerance. To further identify the genes that functionally contributed to the tolerance of the lethal level of hypoxia (5% O_2_), a mutagenesis screen strategy was applied. We first focused on those genes for which single *P*-element insertion *D. melanogaster* lines were available from common *Drosophila* stock centers. One hundred and forty six lines with single *P*-element insertion within or around 82 down-regulated genes and 21 lines with *P*-element insertion within or around 10 up-regulated genes were obtained from *Drosophila* Stock Centers (Table S2). Embryos from each *P*-element line were collected and cultured directly in 5% O_2_ (*no adaptation*) to examine the role of each gene in hypoxia tolerance of *D. melanogaster*. Of interest, most (∼75%) available *P*-element insertion lines representing the up-regulated genes (e.g. *CG1600*, *CG30492*, *GstE1*, *Hph*, and *th*; Table S2) did not survive this severe hypoxic condition. This suggests that most of the up-regulated genes tested by *P*-elements seem to be important for hypoxia tolerance. Out of the 82 down-regulated genes tested, 26 had more than one *P*-element allele available. We then focused mostly on these down-regulated genes and their *P*-elements and performed a series of experiments on them. Six of these 26 genes had multiple *P*-element alleles that showed a remarkably greater survival (3–10 fold increase) as compared to *yw* or any of the parental lines (Figure 4; Table S2). As showed in Figure 5, the transcripts of all alleles were, indeed, down-regulated. In addition, to further confirm that survival in these severe O_2_ conditions was related to the *P*-element insertion in these particular genes, we excised the *P*-element alleles of one gene, namely *sec6*, and found that the precise excision lines had less than 10% of eclosion in 5% O_2_ (Figure 6), a level that is similar to *yw* and naive controls. Therefore, the precise excision of these *P*-elements reversed the hypoxia tolerance phenotype. The sec6 gene encodes a protein that is homologous to a mammalian sec6 protein, and it is predicted to be involved in synaptic vesicle recycling [26]. This presents the first evidence that this gene is involved in conferring hypoxia tolerance in *Drosophila*, possibly through regulation of neurotransmitter release.

These gene families obtained from the microarrays are interesting with respect to the phenotype of hypoxia tolerance and selection since some genes would not have been necessarily predicted. That anti-bacterial peptides were up-regulated in AF is remarkable as such genes are not generally associated with a hypoxia phenotype. On the other hand, protein phosphorylation and dephosphorylation are so ubiquitous and so fundamental to many cellular processes that it is not surprising that kinases or phosphatases are observed to be up- or down-regulated in our microarrays. Previous data have indeed implicated such enzymes in tissue protection during hypoxia [27], [28], [29], [30], [31], [32]. Furthermore, metabolic enzymes such as lipases were down-regulated and it is interesting to speculate that, since this down-regulation has also been found in hypoxia in mammals, the lipase has an important role to play in the accumulation of triglycerides, which, in turn, help in the hypoxia tolerant phenotype [33], [34], [35].

Although the exact molecular mechanisms underlying hypoxia tolerance are currently unknown, it seems that the experimental selection has favored the genetic inheritance of pathways that are important in cell signaling, cell cycle and cell fate determination. For example, gene *lin19* encodes a protein component of SCF ubiquitin ligase complex that is involved in regulating cell cycle and cell proliferation [36]. Interestingly, *Cul1*, the mammalian homologue of *lin19*, encodes a protein that belongs to the Skp1-Cdc53/Cul1-F-box (SCF)-like protein complex which targets specific proteins for ubiquitination and proteolysis under the regulation of pVHL, and the pVHL targeted proteins include transcription factor regulators such as IκB [37].

It is important to emphasize that the disparity in the functions of these genes likely reflects the complex physiological regulation of hypoxic responses in tissues. From our results and the function of the characterized genes obtained (i.e., *Best1, br, CG7102, dnc, lin19* and *sec6*), we found that these genes encode proteins that play a role not only in ubiquitination (e.g. *lin19*) [36], [37] but also in transcriptional regulation (e.g. *broad*) [38], [39], in signal transduction pathways (e.g. *dunce*) [40] and membrane transport of ions or neurotransmitters (e.g. *sec6* and *Best1*) [26], [41]. However, what is remarkable to note is that our data provide evidence, in spite of the complexity of the phenotype of hypoxia tolerance, that *single* genes make a sizeable difference in this phenotype. It is completely possible, however, that such single genes, as has been shown for the HIF-1 transcription factor [2], are master switches. Whether one or more of the genes obtained in this study is another “switch” that can activate or inactivate a large number of genes that are relevant to the hypoxia tolerance is unknown at present.

**In summary**, we have succeeded in selecting for flies that can live perpetually at extremely low O_2_ (5% and 4% O_2_), levels that are lethal in naïve flies. These O_2_ levels exist in atmospheric environments of ∼2,000 and ∼4,000 meters higher than Mt. Everest, respectively, and indeed represent severe conditions. This ability to survive at this extreme “altitude” has now become an inherited trait in the AF flies, a result of a long term experimental selection. The down-regulation of the six genes, *Best1, br, CG7102, dnc, lin19* and *sec6,* seem to play a crucial role in hypoxia tolerance and survival in extreme low O_2_ conditions. In spite of the fact that we elicited the importance of these genes using an experimental selection protocol over generations, these genes may also be critical in hypoxia tolerance in physiological or pathological conditions that are characterized by low O_2_ over shorter periods of time. The fact that inhibition of gene activity in this model system leads to a remarkably higher tolerance to extreme low O_2 _environments implies that inhibition of the mammalian homolog of the candidate genes in mammals may also be an avenue toward ameliorating the effects of hypoxia.

## Supporting Information

Table S1 List of Significantly Altered Genes
(0.08 MB XLS)

Table S2 Eclosion rate of P-element alleles in hypoxic condition
(0.04 MB XLS)

Table S3 List of Specific Primers for sqRT-PCR
(0.03 MB DOC)

Figure S1 The eclosion rate of individual parental lines under 5% of O_2_. The measured eclosion rates ranged from 0 to 28 percent (4.7% in average) demonstrating significant levels of functional variability among these lines with respect to the ability to survive hypoxic condition (ANOVA, p = 0.022).
(1.46 MB TIF)

Figure S2 The recovery time of individual parental lines from anoxic stupor. Recovery time from anoxic stupor of each individual parental line was measured using 5 to 6 days old male adult flies, the recovery time ranged from 319 to 515 seconds demonstrating a significant genetic variation among these lines (ANOVA, p<0.0001).
(1.84 MB TIF)
