# Supplementary material for: Experimental Selection for Drosophila Survival in Extremely Low O2 Environment
Source: PLoS One. 2007 May 30;2(5):e490. doi: 10.1371/journal.pone.0000490 (PMC1871610; doi:10.1371/journal.pone.0000490)
Supplement: Table S3 — List of Specific Primers for sqRT-PCR (0.03 MB DOC) [file pone.0000490.s003.doc]

**Table S3.** List of Specific Primers for sqRT-PCR

| **Symbol** | **Gene Name** | **Primer Sequence** |
| --- | --- | --- |
| Best1 | Bestrophin 1 | Forward: AATGATCCACGTCCCAATGT  Reverse: CTCCGATTTCATCGTCGAAT |
| br | broad | Forward: ATCCAACACACACACGAGGA  Reverse: AGTGCTGTGTGTCGTCCATC |
| CG7102 | CG7102 | Forward: TCTGCTTGGAAGAGGAGGAA  Reverse: GCTGGATTCAAGGCAAAGAG |
| dnc | dunce | Forward: CAGCAAATCCAACAGCTTCA  Reverse: GGCCGACTTGGAGATCAATA |
| lin19 | Lin-19-like | Forward: AGCGCCGAATTAACATCAAC  Reverse: ACAGCCCCTGCAGAAGTCTA |
| sec6 | sec6 | Forward: CCCTAAAGCGTCACTTCGAG  Reverse: CACTCCTGCTTCTCCGTTTC |
