# Supplementary figures and images for: Experimental Selection for Drosophila Survival in Extremely Low O2 Environment
Source: PLoS One. 2007 May 30;2(5):e490. doi: 10.1371/journal.pone.0000490 (PMC1871610; doi:10.1371/journal.pone.0000490)

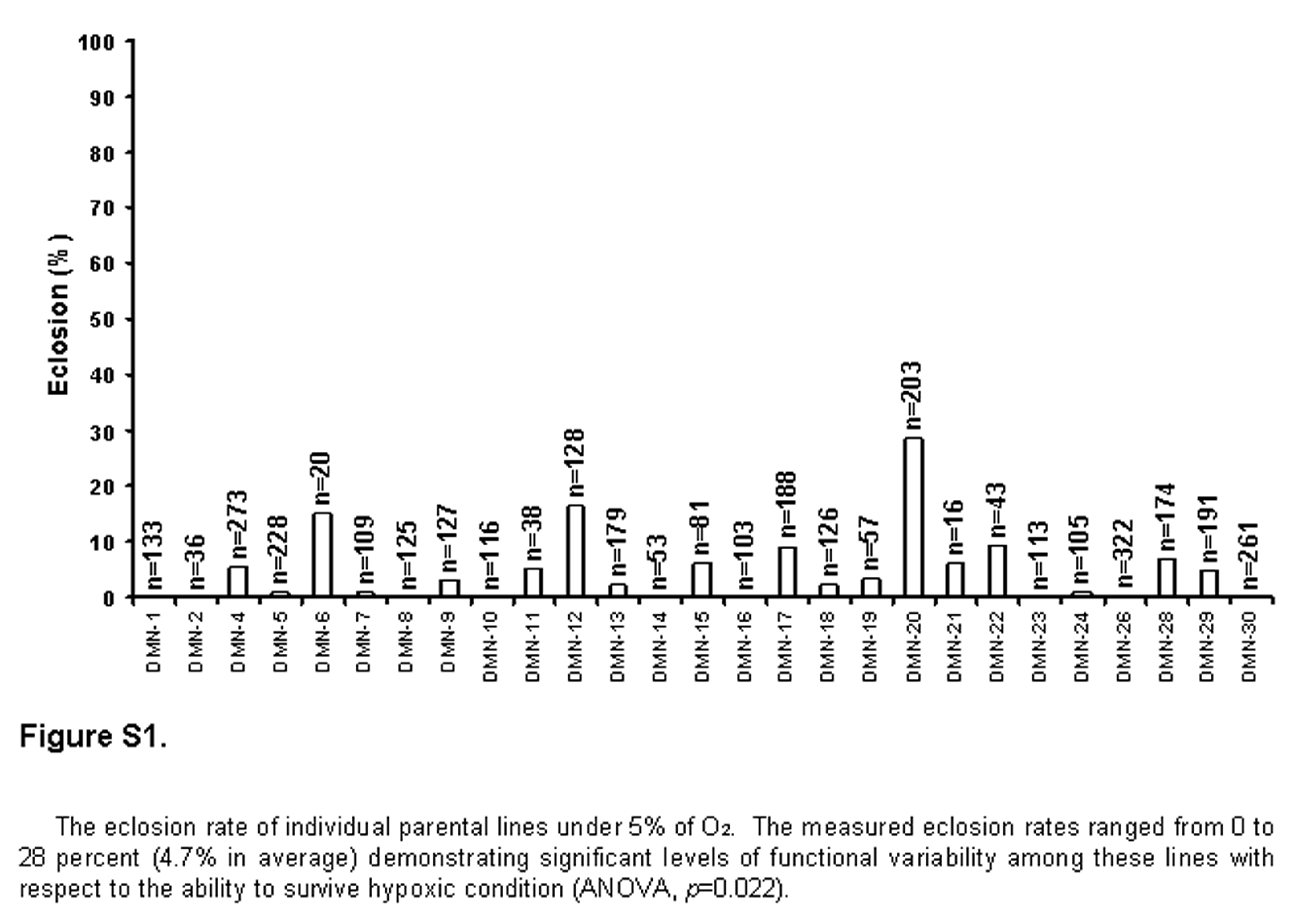

Supplement: Figure S1 — The eclosion rate of individual parental lines under 5% of O2. The measured eclosion rates ranged from 0 to 28 percent (4.7% in average) demonstrating significant levels of functional variability among these lines with respect to the ability to survive hypoxic condition (ANOVA, p = 0.022). (1.46 MB TIF) [file pone.0000490.s004.tif]

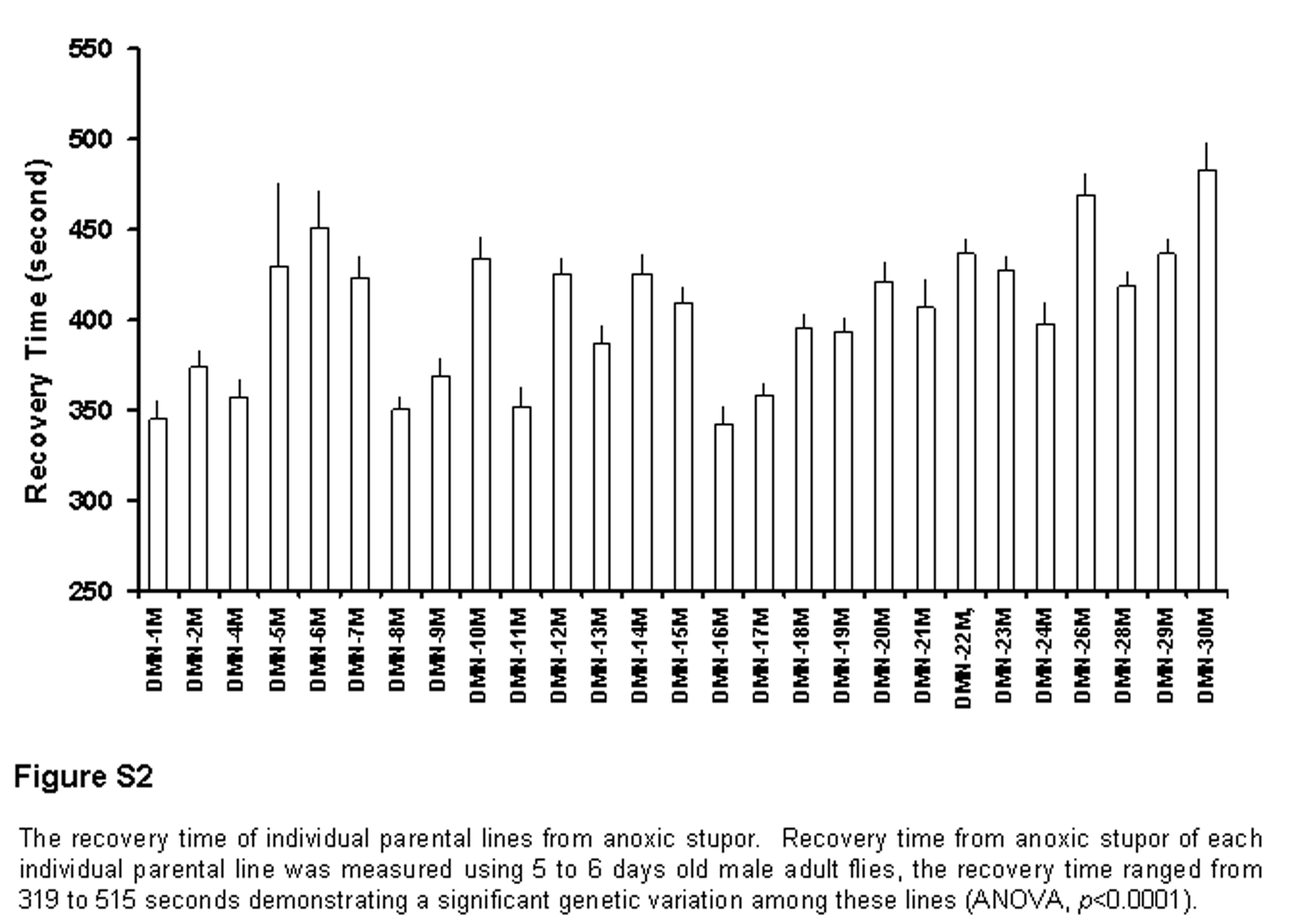

Supplement: Figure S2 — The recovery time of individual parental lines from anoxic stupor. Recovery time from anoxic stupor of each individual parental line was measured using 5 to 6 days old male adult flies, the recovery time ranged from 319 to 515 seconds demonstrating a significant genetic variation among these lines (ANOVA, p<0.0001). (1.84 MB TIF) [file pone.0000490.s005.tif]
